# Drug Resistant Urinary Isolates of Pseudomonas Aeruginosa and Acinetobacter Species

**DOI:** 10.4103/0974-777X.68547

**Published:** 2010

**Authors:** Jyoti Sharma, Neelam Gulati, Jagdish Chander

**Affiliations:** *Department of Microbiology, Dr. H. S. J. Institute of Dental Sciences and Hospital, Panjab University, Chandigarh, India*; 1 *Department of Microbiology, Government Medical College and Hospital, Chandigarh, India*

Sir,

The emergence of antibiotic resistance and its spread has been a serious challenge to the clinicians. Penicillinase represents the infancy of antibiotic resistance, which was followed by penicillin-inactivating enzymes. Recently the focus of clinicians has shifted to non-lactose-fermenting gram-negative pathogens responsible for hospital-acquired infections (HAI).[1] Further, if we talk about intensive care units (ICUs), *Acinetobacter* sp. and *Pseudomonas* sp. are frequently encountered.[2] *Acinetobacter* has emerged as an important and problematic human pathogen as it is the causative agent of several types of infections, including pneumonia, meningitis, septicemia and urinary tract infections (UTI). It is ranked second after *Pseudomonas* among the nosocomial, aerobic, nonfermentative, gram-negative bacilli pathogens. Isolates from both the genera are multidrug resistant and sometimes also exhibit pan resistance. The aim of our study was to find the prevalence of multidrug resistance and pan-drug resistance among gram-negative uropathogens.

The isolates isolated from hospitalized patients at Government Medical College and Hospital, Chandigarh, were collected in the Department of Microbiology from January 2007 to June 2007. The isolates were identified using routine conventional methods based upon microscopic findings, colony morphology and enzymatic characters. Routine antibiotic sensitivity testing was done by disc-diffusion method according to the recommendations of Clinical and Laboratory Standard Institute (CLSI).[3]

The isolates were defined as multidrug-resistant (MDR) phenotypes if they were resistant to more than two classes of antibiotics, cephamycin, aminoglycoside, carbapenem and fluoroquinolones.[4] The isolates were considered to be pan-drug–resistant (PDR) phenotypes if they showed diminished susceptibility to almost all the antibiotics recommended for empirical therapy.[4]

A total of 4912 urine samples were received in the department within the period of 6 months. Out of these, 1409 samples grew pathogens. The details of different bacterial species encountered in urine samples are given in Table 1. The most frequent organism was E. *coli*, followed by K. *pneumoniae*. *Candida* sp. was the commonest fungal isolate. Table 2 depicts the distribution of MDR and PDR isolates amongst gram-negative urinary pathogens. Maximum numbers of MDR and PDR isolates were observed in P. *aeruginosa* (72%); followed by *Acinetobacter* sp., where the value reached up to 62%. The frequency of multidrug resistance and pan-drug resistance in K. *pneumoniae* was 49%; and in E. *coli*, 28%.

**Table 1:** Details of urinary isolates investigated in the study

Group	Bacteria	Number (N)
Gram-negative bacilli	*E. coli*	629
	*K. pneumoniae*	160
	*P. aeruginosa*	92
	*Acinetobacter* spp.	67
Gram-positive cocci	*Enterococcus*	115
	*Staphylococcus*	84
	*Streptococcus*	18
Fungi	Candida	119
Mixed type 1	Two bacterial isolates	37
Mixed type 2	One fungal + one bacterial isolates	19
Miscellaneous		69

**Table 2:** Distribution of MDR and PDR isolates amongst gram-negative bacteria

Bacteria	MDR	PAN resistant	Total (*n*)	Frequency (*n*/N)
*E. coli*	170	8	178	28%
*K. pneumoniae*	71	8	79	49.3%
*P. aeruginosa*	24	42	66	71.7%
*Acinetobacter* spp.	27	15	42	62.6%
Total	292	73	365	38.5%

Foot notes: MDR (multidrug-resistant), PDR (pan-drug–resistant), (*n*=948)

Amongst all isolates, MDR and PDR isolates were mainly distributed amongst gram-negative bacteria, specifically focused at P. *aeruginosa* followed by *Acinetobacter* (71.7% and 62.6%, respectively). P. *aeruginosa* and Acinetobacter have in some cases expressed resistance to all clinically available compounds. P. aeruginosa possesses more tools for defying the activity of antimicrobial agents than virtually any other microorganism.[5] Like P. *aeruginosa*, *Acinetobacter* has been found to express a variety of different resistance mechanisms, including production of aminoglycoside-modifying enzymes, ESBLs and carbapenemases, as well as through changes in outer membrane proteins, penicillin-binding proteins and topoisomerases.[6]

During the last decade, efforts to combat multidrug-resistant microorganisms mainly focused on gram-positive bacteria. Since the number of reports on infections caused by gram-negative microorganisms, for which no adequate therapeutic options exist, is increasing, it is time to intensify attention towards gram-negative resistance.

We must remain mindful that indiscriminate use of any antibacterial agent will inevitably lead to resistance. As such, continued emphasis on effective infection-control measures and judicious and parsimonious use of available antimicrobial agents will remain mainstays in any reasonable strategy to maximize the usefulness of available antimicrobial agents.
